# The New Era for Reno-Cardiovascular Treatment in Type 2 Diabetes

**DOI:** 10.3390/jcm8060864

**Published:** 2019-06-17

**Authors:** Clara García-Carro, Ander Vergara, Irene Agraz, Conxita Jacobs-Cachá, Eugenia Espinel, Daniel Seron, María José Soler

**Affiliations:** 1 Nephrology Research Group, Vall d’Hebron Research Institute (VHIR), Nephrology Department, Hospital Universitari Vall d’Hebron, Universitat Autònoma de Barcelona, 08035 Barcelona, Spain; clara.garcia@vhebron.net (C.G.-C.); vergara.ander@gmail.com (A.V.); iagraz@vhebron.net (I.A.); eespinel@vhebron.net (E.E.); dseron@vhebron.net (D.S.); 2 Red de Investigación Renal (REDINREN), Instituto Carlos IIIFEDER, 28029 Madrid, Spain

**Keywords:** diabetes, diabetic kidney disease, reno-cardiovascular protection, sodium-glucose co-transporter 2 inhibitors, glucagon-like peptide-1 receptor agonists, dipeptidyl peptidase 4 inhibitors

## Abstract

Diabetic kidney disease (DKD) is the leading cause of end-stage renal disease in the developed world. Until 2016, the only treatment that was clearly demonstrated to delay the DKD was the renin-angiotensin system blockade, either by angiotensin-converting enzyme inhibitors or angiotensin receptor blockers. However, this strategy only partially covered the DKD progression. Thus, new strategies for reno-cardiovascular protection in type 2 diabetic patients are urgently needed. In the last few years, hypoglycaemic drugs, such as sodium-glucose co-transporter 2 inhibitors and glucagon-like peptide-1 receptor agonists, demonstrated a cardioprotective effect, mainly in terms of decreasing hospitalization for heart failure and cardiovascular death in type 2 diabetic patients. In addition, these drugs also demonstrated a clear renoprotective effect by delaying DKD progression and decreasing albuminuria. Another hypoglycaemic drug class, dipeptidyl peptidase 4 inhibitors, has been approved for its use in patients with advanced chronic kidney disease, avoiding, in part, the need for insulinization in this group of DKD patients. Studies in diabetic and non-diabetic experimental models suggest that these drugs may exert their reno-cardiovascular protective effect by glucose and non-glucose dependent mechanisms. This review focuses on newly demonstrated strategies that have shown reno-cardiovascular benefits in type 2 diabetes and that may change diabetes management algorithms.

## 1. Introduction

Diabetic kidney disease (DKD) is the first cause of chronic kidney disease, leading to premature death and end-stage renal disease (ESRD) in the developed and developing world. In response, multiple potential therapeutic agents have been tested, focusing on the treatment of hyperglycaemia and hypertension, mainly directed at the renin-angiotensin system blockade [1,2,3,4]. However, these therapies only partially delay the progression of DKD to ESRD, so there is an urgent need for additional effective treatments. In this context, sodium-glucose co-transporter 2 (SGLT2) inhibitors and the glucagon-like peptide-1 receptor agonists (GLP-1RAs) have recently emerged as new potential strategies for both diabetic type 2 and 1 patients [5,6,7].

The SGLT2 is expressed in the proximal tubule of the kidneys and is responsible for 90% of renal glucose reabsorption. SGLT2 inhibitors promote the urinary excretion of glucose and, consequently, lower blood glucose levels. Interestingly, these drugs scarcely provoke hypoglycaemia, as their effect is not related to beta cell function or modifications in insulin sensitivity [8]. SGLT2 inhibitors were first approved by the U.S. Food and Drug Administration (FDA) in 2013 for patients with type 2 diabetes, and the first study that demonstrated their beneficial effects in terms of delaying DKD progression was published in 2016 [5]. Glucagon-like peptide-1 (GLP-1) is an endogenous incretin peptide released from intestinal L cells in response to ingested nutrients. GLP-1 is rapidly inactivated by the enzyme dipeptidyl-peptidase-4 (DPP-4) and cleared by the kidneys. GLP-1 stimulates pancreatic insulin synthesis and insulin secretion in a glucose-dependent manner, slows gastric emptying, inhibits glucagon release, and promotes satiety. GLP-1 receptor agonists are structurally similar to GLP-1 but resist dipeptidyl peptidase 4 (DDP-4) degradation [8]. Exenatide was the first GLP-1RA approved by the FDA in 2005 for patients with type 2 diabetes, and in 2016, liraglutide was the first that demonstrated beneficial effects in terms of decreasing albuminuria [9]. DPP-4 inhibitors are a class of oral hypoglycaemics that block the DPP-4 enzyme and subsequently neutralise several incretin peptides, including the glucose-dependent insulinotropic polypeptide (GIP) and GLP-1 [8]. Thus, DPP-4 inhibitors increase GLP-1 and reduce blood glucose by inhibiting glucagon release and stimulating insulin secretion. Sitagliptin was the first DPP-4 inhibitor approved by the FDA in 2006, followed by linagliptin, saxagliptin, and alogliptin. More recently, in 2019, linagliptin demonstrated beneficial effects in reducing the progression of albuminuria in DKD [10].

In this review, we describe the new strategies for reno-cardiovascular protection in patients with type 2 diabetes and their potential mechanisms. We cover the most important studies focused on the renoprotection exerted by SGLT2 inhibitors, GLP-1RAs, and DPP-4 inhibitors in DKD patients.

## 2. Classical Pharmacological Reno-Cardiovascular Approaches in Diabetes

Patients with diabetes have a higher prevalence of cardiovascular morbidity and mortality compared to the general population [11]. It is well known that diabetes is associated with accelerated atherosclerosis, affecting the coronaries, which increases the risk for myocardial infarction, heart failure, and may cause diabetic cardiomyopathy independent of coronary artery disease, hypertension, and valvular complications [12]. According to some authors, during the early stages of diabetes, there is an increase in plasma renin activity, mean arterial pressure, and renal vascular resistance [13], suggesting that renin-angiotensin-aldosterone system (RAAS) activation plays a major role in the development of cardiovascular disease (CVD) [14]. Therefore, angiotensin-converting enzyme inhibitors (ACEi) and angiotensin II receptor blockers (ARB) have been, for many years, the first line therapy for secondary CVD prevention in patients with diabetes [15].

In the late 80’s to early 90’s, an ACEi, enalapril, demonstrated its effectiveness in reducing mortality in patients with heart failure [16,17]. Later, the heart outcomes prevention evaluation (HOPE) trial included subjects with high cardiovascular risk, such as diabetic patients, and demonstrated that another ACEi, ramipril, significantly reduced the rates of death, myocardial infarction, and stroke in patients with vascular disease or diabetes [14]. Similarly, the losartan intervention for endpoint reduction (LIFE) trial showed that Losartan was more effective than Atenolol in reducing cardiovascular morbidity and mortality in patients with hypertension, diabetes, and ventricular hypertrophy [17]. In 2001, two seminal studies demonstrated the nephroprotective effect of RAAS blockades in patients with type 2 diabetes [2,3]. A subsequent metanalysis supported the use of ACEi in patients who have diabetic kidney disease (DKD) with significant albuminuria [18]; however, the beneficial effect of RAAS inhibition as a primary prevention in diabetic patients has not been demonstrated. The beneficial effect of RAAS blockade in patients with advanced chronic kidney disease (estimated glomerular filtration rate (eGFR) < 30 mL/min/1.73 m^2^) is unknown, given the fact that those patients have been systematically excluded from clinical trials [1,19].

After the previously mentioned results, and based on preliminary studies demonstrating an added effect on decreasing albuminuria with a dual RAAS blockade [18], later research was focused on studying the effect of the combination of ACEi and ARB in high risk DKD patients [20]. During this period, the ongoing telmisartan alone and in combination with ramipril global endpoint trial. (ONTARGET) evaluated whether the combination of an ACEi (ramipril) with an ARB (telmisartan) was better than the full dose of either drug. This study showed that there was no superiority of the ACEi versus the ARB and that the dual blockade did not confer greater cardiovascular protection. Moreover, the combination of ACEi and ARB increased the risk of adverse events, namely hyperkalemia, hypotensive symptoms, and the over declined eGFR, more than monotherapy [20]. Similar results were obtained from the aliskiren trial in type 2 diabetes using cardio-renal endpoints (ALTITUDE), which compared the effect of a direct renin inhibitor aliskiren to placebo in high-risk type 2 diabetic patients on top of an ACEi or an ARB. The ALTITUDE trial, just like the ONTARGET study, demonstrated that the simultaneous administration of aliskiren with an ACE inhibitor or an ARB should be avoided. The study was halted early because an increased incidence of hypotension, hyperkalemia, renal complications, and non-fatal stroke (HR = 1.25; 95% CI 0.98–1.60; *p* = 0.07) was observed in the aliskiren arm during the follow-up of approximately 2.7 years [21]. Consequently, Novartis (Basel, Switzerland) immediately suspended all promotional and educational programs related to aliskiren and its combinations.

## 3. Reno-Cardiovascular Protection of SGLT2 Inhibition

SGLT2 inhibitors enhance renal glucose excretion by inhibiting renal glucose reabsorption in the renal proximal tubule. Consequently, SGLT2 inhibitors reduce plasma glucose in an insulin-independent manner and improve insulin resistance in diabetes [22]. SGLT2 inhibitors were shown to reduce glycated haemoglobin (HbA1c) by approximately 0.6%–1.2%, with a lower rate of hypoglycaemia [23]. Four recent major trials have shown that SGLT2 inhibitors are superior to other anti-diabetic medications in the prevention of cardiovascular events and renal protection [5,24,25,26]. The empagliflozin cardiovascular outcome event trial in type 2 diabetes mellitus patients (EMPA REG OUTCOME ) was the first clinical trial examining the effects of empagliflozin compared to placebo on cardiovascular morbimortality in patients with type 2 diabetes and at a high risk for cardiovascular events [27]. EMPA REG included 7020 patients, all of them with established cardiovascular disease and an eGFR ≥ 30 mL/min/1.73 m^2^, randomly assigned to receive empagliflozin at −10 or 25 mg or a placebo on top of standard care. Death from cardiovascular causes, non-fatal myocardial infarction, or non-fatal stroke was decreased in the empagliflozin group (10.5%) compared to the placebo group (12.1%) (HR 0.86; 95% CI 0.74–0.99; *p* = 0.04). Interestingly, this benefit was higher in older patients (>65 years) and with HbA1c ≤ 8.5%. Empagliflozin more significantly decreased cardiovascular death (3.7% vs. 5.9%) and death from any cause (5.7% vs. 8.3%) compared to placebo (HR 0.62; 95% CI 0.49–0.77; *p* < 0.001 and HR 0.68; 95% CI 0.57–0.82, *p* < 0.001), as well as hospitalization for heart failure (HR 0.65; 95% CI 0.50–0.85; *p* = 0.002). HbA1c levels were similar in both groups. In contrast, in previous studies, empagliflozin was associated with a decrease in HbA1c levels in patients with type 2 diabetes [28,29]. In the EMPA REG study, patients receiving empagliflozin showed a decrease in weight, waist circumference, uric acid level, blood pressure, and increased cholesterol levels. 2250 out of 7020 patients (32%) included in the EMPA REG OUTCOME trial had chronic kidney disease (eGFR < 60 mL/min/1.73 m^2^ and/or macroalbuminuria) [5], and 1896 patients (27%) presented microalbuminuria and eGFR ≥ 60 mL/min/1.73 m^2^. Treatment with empagliflozin showed benefits in terms of incident or worsening nephropathy (12.7% vs. 18.8% in placebo group) (HR 0.61; 95% CI 0.53–0.70; *p* < 0.001) and progression to macroalbuminuria (11.2% vs. 16.2%) (HR 0.62; 95% CI 0.54–0.72; *p* < 0.001). Patients receiving empagliflozin demonstrated a decrease in the doubling of serum creatinine compared to the placebo group (HR 0.56; 95% CI 0.39–0.79; *p* < 0.001), as well as a lower rate of renal replacement therapy initiation (0.3% vs. 0.6%) (HR 0.45; 95% CI 0.21–0.97; *p* = 0.04) (see Table 1). eGFR decreased in the empagliflozin group within the first weeks of treatment, but at the end of follow up, eGFR remained stable with empagliflozin compared to a decrease in the placebo group [30].

The Canvas Program studied the cardiovascular and renal effects of canagliflozin (100 mg or 300 mg) versus placebo in 10,142 type 2 diabetic patients with a previous history of cardiovascular disease or two or more cardiovascular risk factors and eGFR > 30 mL/min/1.73 m^2^. The mean eGFR was 76.5 ± 20.5 mL/min/1.73 m^2^; 22.6% presented microalbuminuria and 7.6% presented macroalbuminuria. Canagliflozin administration significantly decreased HbA1c levels, as well as blood pressure and body weight. In the canagliflozin group, deaths from cardiovascular cause, non-fatal myocardial infarction, and non-fatal stroke were significantly decreased by 14% (26.9 vs. 31.5 patients, with an event per 1000 patient-years) [24]. Regarding renal outcomes, canagliflozin reduced the risk of the composite outcome of a sustained 40% reduction in eGFR, renal replacement therapy initiation, and death from renal causes. Canagliflozin reduces albuminuria progression (HR 0.73; 95% CI 0.67–0.69), and the regression of albuminuria occurred more frequently in canagliflozin treated patients (HR 1.7; 95% CI 1.51–1.91).

The dapagliflozin effect on cardiovascular events (DECLARE-TIMI 58) evaluated the effects of dapagliflozin (SGLT2 inhibitor) versus placebo on cardiovascular and renal outcomes in patients who had, or were at risk for, cardiovascular disease. Eligible patients were older than 40 years old, had type 2 diabetes, a history of cardiovascular disease or more than two classical cardiovascular risk factors, and an eGFR ≥ 60 mL/min/1.73 m^2^. The study included 17,160 patients, 40.6% with cardiovascular disease and 59.4% with multiple cardiovascular risk factors. The mean eGFR was 85.2 mL/min/1.73 m^2^ and only 7% of patients had an eGFR <60 mL/min/1.73 m^2^. Dapagliflozin significantly decreased HbA1c levels, body weight, and blood pressure [25]. There were no differences in cardiovascular death, myocardial infarction, and ischemic stroke between the two groups. Dapagliflozin reduced the risk of this composite outcome by 17% due to a significantly lower rate of hospitalization for heart failure in the dapagliflozin group (HR 0.73; 95% CI 061–0.88). It also reduced the composite renal outcome (a decrease of 40% or more in eGFR, end-stage renal disease, or death from renal or cardiovascular cause) risk by 24% (HR 0.76; 95% CI 0.67–0.87). Interestingly, the renoprotection observed with dapagliflozin was independent of the presence of established CVD [25].

The renoprotective effect of SGLT2 inhibition has become evident in the last 3 years. However, until now its use has been limited to patients with eGFR > 45 mL/min/1.73 m^2^ (Table 2). A recent paper published by Vlado Perkovic clearly demonstrated the beneficial effect of canagliflozin in patients with moderate–advanced DKD (CREDENCE trial) [26]. This trial studied the effect of canagliflozin compared to placebo in patients with eGFR = 30–90 mL/min/1.73 m^2^ and a urinary albumin-to-creatinine ratio > 300–5000 mg/g creatinine, receiving a stable dose of an ACE or ARB. It included 4401 patients with a mean eGFR of 56.2 mL/min/1.73 m^2^ and a mean albumin-to creatinine ratio of 927 mg/g. Of note, 31% of patients had an eGFR < 45 mL/min/1.73 m^2^. The trial was halted early because the number of primary outcome events in the placebo group required to trigger analysis was reached sooner than estimated. Canagliflozin decreased the risk of end-stage kidney disease, doubling of the serum creatinine level, or renal or cardiovascular death by 30% compared to placebo [26]. Patients in the canagliflozin group also showed a 30% reduction in the risk of cardiovascular death or hospitalization for heart failure (HR 0.69; 95% CI 0.57–0.83; *p* < 0.001), a 20% reduction of cardiovascular death, myocardial infarction or stroke (HR 0.80; 95% CI 0.67–0.95), and a 29% reduction of hospitalization for heart failure (HR 0.61; 95% CI 0.47–0.80). Canagliflozin group showed a decreased eGFR slope compared with the placebo group (−3.19 ± 0.15 vs. −4.71 ± 0.15 mL/min/1.73 m^2^ per year), which means a difference of 1.52 mL/min/1.73 m^2^ per year. Rates of adverse events were similar in both groups. In conclusion, the use of canagliflozin in type 2 diabetic patients with established kidney disease on top of renin-angiotensin system blockade was safe and decreased the risk of kidney failure and cardiovascular events [26].

## 4. Reno-Cardiovascular Protection of GLP1 Receptor Agonists

GLP-1RAs are glucagon-like peptide-1 (GLP-1) molecule analogues. GLP-1 is an incretin secreted by intestinal enteroendocrine L-cells in response to food intake, which increases insulin secretion in a glucose-dependent manner [31]. GLP-1 Ras have been shown to improve glycaemic control and reduce glycated haemoglobin (HbA1c) by approximately 1%–1.5% in short-term treatments with a lower rate of hypoglycaemia [9,32]. The reduction of hypoglycaemic events is important in diabetic patients with chronic kidney disease (CKD), where GLP-1RAs have proven to be safe while many other antihyperglycemic drugs require dose adjustment as insulin or are simply contraindicated (Table 2). These new drugs have also been shown to reduce weight during treatment, which seems to be a class effect and an interesting outcome when it comes to treating overweight type 2 diabetic patients [9,32,33,34]. However, their most significant impact is that GLP-1RAs were demonstrated to reduce major adverse cardiovascular events (MACEs), liraglutide, semaglutide, and albiglutide [9,33,35], and delay DKD progression liraglutide, semaglutide, and dulaglutide [6,32,33].

In the liraglutide effect and action in diabetes: evaluation of CV outcome results (LEADER) trial, Liraglutide was shown to decrease three-point MACEs and death from cardiovascular causes in a 3.8 year follow-up when compared to the placebo added to standard care (HR 0.87; 95% CI 0.78–0.97; *p* < 0.001) [9]. Moreover, death from any cause was lower in the Liraglutide group and, although there were no statistically significant differences in the occurrence of myocardial infarction or stroke between both groups, there was a trend toward a reduced incidence of both events. In the subgroup analyses, these protective effects were more evident in patients with kidney disease and an eGFR rate below 60 mL/min/1.73 m^2^, as well as in patients with cardiovascular disease at baseline. In concordance, semaglutide also reduced the MACE composite outcome when compared to placebo during a follow-up period of 2.1 years in the trial to evaluate cardiovascular and other long-term outcomes with semaglutide in subjects with type 2 diabetes (SUSTAIN-6) (HR 0.74; 95% CI 0.58–0.95; *p* < 0.001) [33]. There was a significant reduction in the incidence of non-fatal stroke and a non-significant trend for a lower incidence of non-fatal myocardial infarction in the semaglutide group. However, and in contrast to the results displayed in the LEADER trial, the rates of death from cardiovascular causes, and death from any cause, were similar in both groups. This could be, in part, explained by the larger number of patients recruited in LEADER and the longer observation time. In the randomized, double-blind, placebo-controlled trial of the effect of albiglutide on major cardiovascular events in patients with type 2 diabetes mellitus (HARMONY), albiglutide also reduced the incidence of three-point MACEs compared to placebo during a follow-up of 1.6 years (HR 0.78; 95% CI 0.68–0.90; *p* < 0.0001) [35]. This decrease was mainly driven by a reduction in the incidence of myocardial infarction, but rates of stroke and death from cardiovascular causes were similar in both treatment groups.

Conversely, other GLP-1RAs did not demonstrate clear cardiovascular benefits. In the evaluation of lixisenatide in acute coronary syndrome (ELIXA) trial, lixisenatide exhibited non-inferiority when compared to a placebo added to standard care during a 25 month follow-up in patients with established cardiovascular disease, but did not reduce the incidence of the composite cardiovascular outcome [36]. Nonetheless, all patients included in the ELIXA were at high cardiovascular risk, with a recent previous history of myocardial infarction or unstable angina, while 81.2% of patients in LEADER had established cardiovascular disease, without previous ischemic heart disease [9], and 60.5% in SUSTAIN-6 [33], and 71% of patients in HARMONY [35] had a previous history of ischemic heart disease. Another trial, the exenatide study of cardiovascular event lowering (EXSCEL), showed a non-significant trend to reduce the incidence of three-point MACEs compared to placebo, where 73.1% of the trial population had previous cardiovascular disease [34].

When it comes to evaluating the renoprotective effects of GLP-1RAs, trials usually examine a composite outcome of new-onset persistent macroalbuminuria, persistent doubling of serum creatinine, end-stage renal disease, or death due to renal causes. A secondary renal outcome analysis of LEADER showed a reduced incidence of this composite outcome in patients receiving liraglutide (HR 0.78; 95% CI 0.67–0.92; *p* = 0.003) [6]. This reduction was mainly driven by a lower incidence of persistent macroalbuminuria, but there were no differences in the doubling of serum creatinine, end-stage renal disease, or death due to renal causes between the groups. New-onset microalbuminuria incidence was lower in the treated group. The effect of Liraglutide appeared to be independent in a subgroup analysis by baseline albuminuria or eGFR. A slightly slower decline of eGFR was also observed in the treated group. In the SUSTAIN-6 trial, semaglutide also reduced the secondary composite renal outcomes of new or worsening nephropathy (HR 0.64; 95% CI 0.46–0.88; *p* = 0.005) [33]. Recently, secondary renal outcomes analysis of the dulaglutide versus insulin glargine in patients with type 2 diabetes and moderate-to-severe chronic kidney disease (AWARD-7) trial revealed that dulaglutide therapy in patients with moderate-to-severe CKD (stages 3 and 4) reduced the glomerular filtration rate decline in the short-term when compared to insulin glargine during a 1 year follow-up [32]. This finding was especially significant in participants who seemed to have a more severe disease with a baseline urine albumin-to-creatinine ratio (UACR) higher than 300 mg/g. In addition, albuminuria reduction was more pronounced in this subgroup (AWARD-7). Interestingly, the AWARD-7 is an randomized clinical trial (RCT), where dulaglutide was compared to insulin glargine, and both treatments were combined with insulin lispro. There were little to no differences in glycaemic control between groups, which may indicate that protective renal effects could be mediated by other mechanisms related to this pharmacological class [32]. Whether cardiovascular protection is a class effect or a specific outcome of certain GLP-1RAs is still controversial and future trials like the dulaglutide and cardiovascular outcomes in type 2 diabetes (REWIND) will give us more information on this matter. Furthermore, although it seems that GLP-1RAs exert renoprotective effects, specific trials should be designed to evaluate this aspect.

## 5. Combination of SGLT2 Inhibitors and GLP1 Receptor Agonists in Diabetes

Despite their promising cardiovascular and renal protective effects, there are still very few trials that verify the positive outcomes of SGLT2 inhibitors and GLP-1RAs used in combination. In the randomized controlled trial 104-week results—once-weekly exenatide plus once-daily dapagliflozin vs. once-weekly exetanide or dapagliflozin alone (DURATION-8), patients with type 2 diabetes inadequately controlled by metformin were randomly assigned to receive exenatide plus dapagliflozin, exenatide alone, and dapagliflozin alone [37]. During a 28 week follow-up, there was a significant reduction of HbA1c in the combined therapy, compared to exenatide or dapagliflozin, and a considerably higher proportion of patients achieved a glycated haemoglobin ≤7%. Exenatide plus dapagliflozin also exhibited a significant weight reduction compared to dapagliflozin or exenatide alone, and systolic blood pressure (SBP) reduction was slightly higher in the combined treatment. It is worth mentioning that no episodes of hypoglycaemia were described and only one case of ketoacidosis was reported in the exenatide group. In the dulaglutide as add-on therapy to SGLT2 inhibitors in patients with inadequately controlled type 2 diabetes (AWARD-10) trial, two different doses of dulaglutide (0.75 or 1.5 mg) or placebo were assigned to patients previously receiving SGLT2 inhibitors with or without metformin [38]. Dapagliflozin and empagliflozin were the most used SGLT2 inhibitors during the 24 week follow-up. A higher proportion of patients in both dulaglutide groups also achieved the goal of HbA1c ≤ 7%. Only the dulaglutide dose of 1.5 mg provided a significant weight and SBP reduction when compared to placebo. Similar to DURATION-8, the reported adverse effects were mainly gastrointestinal and were more frequently described with high doses of dulaglutide. There were no differences in hypoglycemic events between groups, and the described rates were low. In another recent trial, the superior efficacy of insulin degludec/liraglutide vs. insulin glargine (IGlar U100) as add-on to SGLT2 inhibitors ± oral antidiabetic drug therapy in patients with type 2 diabetes (DUAL IX) trial, patients already receiving a SGLT2 inhibitor with or without other oral antidiabetic drugs were randomized to be treated with an injectable combination of insulin degludec and liraglutide (IDegLira) or insulin glargine (IGlar) alone [39]. After 26 weeks of treatment, the group receiving IDegLira had a significant reduction in HbA1c compared to IGlar (treatment difference −0.36%; 95% CI −0.5, −0.21; *p* < 0.0001). However, no changes in body weight were found with the IDegLira treatment. Hypoglycaemic episodes, although were rarely described, were more frequent when compared to the rates displayed in DURATION-8 and AWARD-10, which may be related to the addition of insulin.

Considering the evidence, the combination of the SGLT2 inhibitor and GLP-1RAs has proven to be safe and well tolerated. The rates of adverse effects are similar to those described in monotherapy and of a mild to moderate intensity. Hypoglycaemic episodes were barely described, although no trials have evaluated their safety in populations with established chronic kidney disease, where there is a higher risk of antidiabetic treatment related hypoglycaemia. Greater reductions of HbA1c, body weight, and blood pressure were observed in combined treatments. Despite these results, cardiovascular outcomes and/or DKD progression with add-on therapy have not been analyzed in the previous trials. Therefore, more studies are needed to evaluate these events.

## 6. Potential Nephroprotective Mechanisms of SGLT2 Inhibitors and GLP1 Receptor Agonists

**Figure 1:**
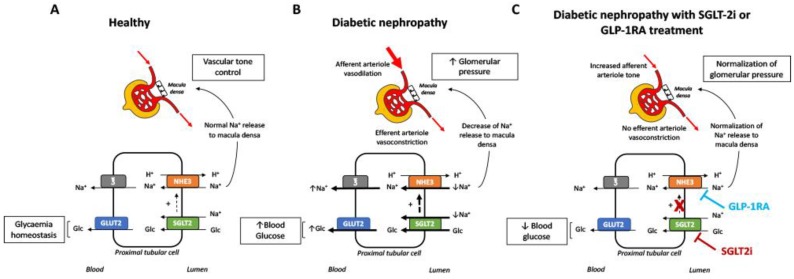
Mechanisms of glucose reabsorption and intraglomerular pressure control in healthy individuals and in diabetic patients. (**A**) In healthy individuals, approximately 90% of glucose is reabsorbed by SGLT2 that is located at the apical membrane of the proximal tubular cells and transported back to the blood stream by the basolateral glucose transporter 2 (GLUT2) transporter. Na^+^ is reabsorbed by the apical transporters SGLT2 (with glucose) and the Na^+^/H^+^ exchanger isoform 3 (NHE3) and returned to the circulation via several basolateral Na^+^ transporters: sodium bicarbonate transporters, Na^+^ channels, and the Na^+^/K^+^ ATPase. Tubular glucose and Na^+^ reabsorption mechanisms contribute to glucose homeostasis and glomerular tone control thanks to the tubuloglomerular feedback controlled by the macula densa. (**B**) In diabetic patients, glucose and Na^+^ reabsorption mechanisms are increased secondary to the hyperfiltration. This fact contributes importantly to the hyperglycaemia and raises the intraglomerular pressure. (**C**) Both SGLT2i and GLP-1RAs produce natriuresis that leads to a decrease of the glomerular pressure. In the case of SGLT2 inhibitors, the natriuretic effect is due to the direct blockade of SGLT2 and the collateral inhibition of NHE3, which has an SGLT2 dependent activity. Regarding GLP-1RAs, these drugs impair only NHE3 activity by an unknown mechanism. Moreover, SGLT2 inhibition contributes to blood glucose level control. ℥: Basolateral Na^+^ transporters.

Blood glucose level and the reduced blood pressure produced by SGLT2 inhibitors and GLP-1RAs have undeniable beneficial effects in terms of cardio-renal protection. However, other independent pathways have also been described and may potentially be relevant to explain their renoprotective effects. Both drugs have natriuretic properties that produce hemodynamic effects on the kidneys. In healthy subjects, glucose and sodium (Na^+^), among other metabolites and electrolytes, are reabsorbed by the tubular cells, mainly in the proximal portion (98% of the total glucose and 67% of the Na^+^). Glucose is absorbed together with Na^+^ by sodium-glucose co-transporters 1 and 2 (SGLT1 and SGLT2) located at the apical membrane of the tubular cells. Although both transporters are expressed in the tubular cells, the major part of the glucose (90%) is reabsorbed by SGLT2 [40,41]. Na^+^ is further reabsorbed by other transporters, of which the Na^+^/H^+^ exchanger isoform 3 is the most important (NHE3) [42]. Glucose is transported to the blood stream by the glucose transporters (GLUT) -mainly GLUT2- [40,41] and Na^+^ via the Na^+^/K^+^ ATPase [42], both located at the basolateral membrane of the tubular cells. This active transport of glucose and Na^+^ contributes importantly to glucose homeostasis and to the maintenance of the intraglomerular tone. In diabetic patients, due to glomerular hyperfiltration, glucose and Na^+^ levels are increased in the lumen of the tubules. In response, the tubular reabsorbing mechanisms are upregulated both by an increase in the activity or expression of the transporters mentioned above [43] and by a translocation of GLUT2 to the apical membrane [44,45]. Therefore, glucose and Na^+^ blood levels rise, contributing to hyperglycaemia and hypertension. In addition, the enhanced reabsorption in the proximal tubule, decreases Na^+^ flow to the distal tubule and activates the macula densa. Both SGLT2 inhibitors and GLP-1RAs increase natriuresis, leading to the restoration of the tubuloglomerular feedback, which results in afferent arteriole vasoconstriction and, finally, in a reduction of the intraglomerular pressure. SGLT2 inhibitors have a more important natriuretic effect than GLP-1RAs, most probably related to the direct blockade of the tubular Na^+^ uptake mediated by SGLT2 and/or the impairment of the Na^+^/H^+^ exchanger isoform 3 (NHE3) activity, which is glucose-dependent and seems to be indirectly blocked by SGLT2 inhibition [46] (see Figure 1). It has recently been described that treatment with empagliflozin decreases NHE3 expression in the kidneys of diabetic otsuka long-evans tokushima Fatty (OLETF) rats, as well as the expression of Na^+^-K^+^-2Cl^−^ cotransporters and epithelial Na^+^ channels, when compared to untreated littermates, suggesting that SGLT2 modulates the Na^+^ reabsorption of several tubular transporters [47]. GLP-1RAs only seem to decrease NHE3 activity [48,49,50], a fact that would explain the difference in the natriuretic potential of these two drugs [51]. It is unclear whether GLP-1RAs inhibit NHE3 activity through interaction with the GLP-1 receptor or extrarenal mechanisms, such as the renin–angiotensin system or atrial natriuretic peptide modulation [52,53].

Interestingly, SGLT2 inhibitors and GLP-1RAs probably have other effects on the kidneys, independent of the glycaemic control and the natriuresis produced by both drugs. As mentioned before, SGLT2 is clearly expressed in the tubular compartment [54]. However, the glomerular cells can also express SGLT2 under protein overload conditions [55], suggesting that this transporter can be upregulated in a non-diabetic context. In addition, SGLT2 is overexpressed in human tubular cells in a culture (HK-2) treated with transforming growth factor beta 1 (TGF-β1). In this model, the effects of TGF-β1 are reverted by empagliflozin most probably by nuclear factor kappa B/toll-like receptor 4 (NF-κB/TLR4) pathway inhibition [56]. Moreover, non-diabetic murine and rat models of kidney injury have shown that treatment with SGLT2 inhibitors decreases kidney fibrosis and inflammation markers [55,57,58]. In a similar way, GLP-1RAs seem to have beneficial effects on the kidneys beyond natriuresis. In a high-fat-diet-induced obesity mice model with renal impairment, liraglutide treatment likely protected subjects from kidney injury through lipid and mitochondrial metabolism regulation via the sirtuin/AMP-activated protein kinase/peroxisome proliferator-activated receptor gamma coactivator 1-alpha (Sirt1/AMPK/PGC1α) pathways [59]. The results obtained in diabetic and non-diabetic experimental models using SGLT2 inhibitors and GLP-1RAs clearly highlight the possibility that these drugs have a direct protective effect on the kidneys.

## 7. Current Role of DPP-4 Inhibitors in Patients with Type 2 Diabetes and CKD

The indications for Dipeptidyl Peptidase 4 Inhibitors in patients with type 2 diabetes and CKD have been analyzed over the last 10 years. In early 2010, diverse studies involving a small number of patients demonstrated the safety and efficacy of an adjusted dose of vildagliptin in patients with advanced CKD in hemodialysis and peritoneal dialysis [60,61]. In agreement with these studies, clinical trials performed with alogliptin and sitagliptin demonstrated the safety and efficacy of these DPP-4 inhibitors in type 2 ESRD patients receiving dialysis [62,63]. Later studies by Laakso et al. showed that no dose adjustment was needed when linagliptin was used in patients with moderate to severe renal impairment [64]. Drug dosage adjustments based on renal function of the currently available DPP-4 inhibitors are depicted in Table 2. Three important randomized clinical trials assessed the protective effect of DPP-4 inhibitors on renal functions. The first published study, the saxagliptin and cardiovascular outcomes in patients with type 2 diabetes mellitus (SAVOR-TIMI 53) trial, demonstrated that treatment with saxagliptin was associated with a reduction in UACR compared with placebo (median observation time, 2.1 years) in patients with T2D at a high cardiovascular risk with diverse baseline renal characteristics, including a substantial number of patients with renal dysfunction and/or albuminuria. Interestingly, decreased UACR in saxagliptin-treated patients seemed to be independent of its effect on glycemia. This was observed in patients with normoalbuminuria, microalbuminuria, and macroalbuminuria, irrespective of their eGFR at baseline. However, the positive effect of saxagliptin on UACR was not accompanied by a reduction of other renal outcomes [65]. The second study, the linagliptin and its effects on hyperglycaemia and albuminuria in patients with type 2 diabetes and renal dysfunction (MARLINA-T2D) trial, was aimed at investigating the glycaemic and renal effects of linagliptin added to the standard-of-care in patients with type 2 diabetes and albuminuria [66]. Previous studies by the same group retrospectively included four completed studies with 217 patients with type 2 diabetes and prevalent albuminuria, randomized to either linagliptin 5 mg/day (*n* = 162) or placebo (*n* = 55). They found that linagliptin significantly reduced albuminuria from baseline by 28%, compared to placebo, after 24 weeks of treatment. Of note is that an effect on albuminuria was already seen after 12 weeks of treatment [67]. In the MARLINA trial, linagliptin improved glycaemic control in type 2 diabetes patients and those with early stages of diabetic kidney disease but did not improve renal damage, as estimated using the surrogate endpoint of albuminuria, although significantly more of the participants in the linagliptin group than those in the placebo group experienced a meaningful improvement in albuminuria [66]. The last recently published clinical trial, the cardiovascular and renal microvascular outcome study with linagliptin (CARMELINA) study, aimed to test the long-term (median observation time, 2.2 years) effect of linagliptin on hard renal outcomes. This study included adults with type 2 diabetes and high cardiovascular and renal risk (74% of patients had prevalent chronic kidney disease, 43% had an eGFR below 45 mL/min/1.73 m^2^, and 15.2% had an eGFR below 30 mL/min/1.73 m^2^). Linagliptin reduced the progression of the albuminuria category (i.e., a change from normoalbuminuria to microalbuminuria/macroalbuminuria or a change from microalbuminuria to macroalbuminuria) by 14% (HR 0.86; 95% CI 0.78–0.95; *p* = 0.003), although the composite renal endpoints of sustained ESRD, death due to renal failure, or a sustained decrease of 50% or more were not different [10].

## 8. Conclusions

Diabetic kidney disease is the leading cause of premature death and end-stage renal disease in the developed world. Until 2016, the only treatment that demonstrated to be able to attenuate DKD was a renin-angiotensin system blockade, either by ACEi or ARB. However, the partial effectiveness of these agents means that new therapeutic strategies are still needed to delay or prevent progression to ESRD. In recent years, the reno-cardiovascular safety profile of SGLT2 inhibitors and GLP-1RAs has been demonstrated [6,9,68,69,70]. These new drug classes offer reno-cardiovascular protective effects in diabetic patients. Thus, cardiologist and nephrologists should consider the administration of SGLT2 inhibitors or GLP-1RAs for renovascular protection in their type 2 diabetic patients. Recent studies published by endocrinologists, nephrologists and/or cardiologist all recommend the use of SGLT2 inhibitors or GLP-1RAs as a second line treatment in type 2 diabetic patients when it is not contraindicated [71,72,73,74].

## Figures and Tables

**Table 1:** Summary of renal outcomes in controlled randomized trials with sodium-glucose co-transporter 2 (SGLT2) inhibitors and glucagon-like peptide-1 (GLP 1) receptor agonists.

Pharmacological Class	SGLT2 Inhibitors				GLP-1 Receptor Agonists	
Trials	EMPA-REG OUTCOME	CANVAS Program	DECLARE-TIMI 58	CREDENCE	LEADER	SUSTAIN-6
**Antidiabetic agent**	Empagliflozin	Canagliflozin	Dapagliflozin	Canagliflozin	Liraglutide	Semaglutide
**Median follow-up (years)**	3.1	2.4	4.2	2.6	3.84	2.1
**Number of patients (n) (active vs. placebo)**	4687 vs. 2333	5795 vs. 4347	8582 vs. 8578	2202 vs. 2199	4668 vs. 4672	1648 vs. 1649
**% Patients with moderate-to-severe renal disease ^a^**	25.9%	NR	7%	59.8%	23.1%	28.5%
**Hazard Ratio (95% CI)**						
**Composite renal outcome**	0.61 (0.53–0.70) ^b^	0.60 (0.47–0.77) ^c^	0.76 (0.67–0.87) ^d^	0.70 (0.59–0.82) ^e^	0.78 (0.67–0.92) ^b^	0.64 (0.46–0.88) ^b^
**New onset of persistent macroalbuminuria**	0.62 (0.54–0.72)	NR	NR	NR	0.62 (0.54–0.72)	NR
**Persistent doubling of serum creatinine**	0.56 (0.39–0.79) ^f^	NR	NR	0.60 (0.48–0.76) ^g^	0.89 (0.67–1.19) ^f^	NR
**Initiation of renal-replacement therapy**	0.45 (0.21–0.97)	NR	NR	0.74 (0.55–1.00)	0.87 (0.61–1.24)	NR
**Death due to renal disease**	NA	NR	NR	NA	1.59 (0.52–4.87)	NR

^a^ Estimated glomerular filtration rate (eGFR) of less than 60 mL/min/1.73 m^2^. Different primary composite outcomes: ^b^ new-onset of persistent macroalbuminuria, persistent doubling of serum creatinine and an eGFR less than 45 mL/min/1.73 m^2^, need for renal-replacement therapy in the absence of a reversible cause or death due to renal disease; ^c^ 40% reduction in the eGFR, need for renal-replacement therapy in the absence of a reversible cause or death due to renal disease; ^d^ ≥40% decrease in eGFR to less than 60 mL/min/1.73 m^2^, new end-stage renal disease or death from renal or cardiovascular causes; ^e^ end-stage kidney disease, persistent doubling of serum creatinine level or death from renal or cardiovascular causes. ^f^ persistent doubling of serum creatinine and an eGFR less than 45 mL/min/1.73 m^2^; ^g^ persistent doubling of serum creatinine level. NA: not applicable because there are less than 10 events reported. NR: not reported.

**Table 2:** New antidiabetic drugs, dipeptidyl peptidase 4 (DDP4) inhibitors, GLP-1 receptors agonists and SGLT2 inhibitors dose and drug indications according chronic kidney disease stage.

	Chronic Kidney Diseases Stages (mL/min/1.73 m^2^)					
	1 (≥90)	2 (60–89)	3a (45–59)	3b (30–44)	4 (15–29)	5 (<15)
**DPP4 inhibitors (oral)**						
Sitagliptin	25–100 mg/day	No dose adjustment		50 mg/day	25 mg/day	
Vildagliptin	50–100 mg/day	No dose adjustment		50 mg/day		
Saxagliptin	2.5–5 mg/day	No dose adjustment		2.5 mg/day	Avoid in Dialysis	
Linagliptin	5 mg/day	No dose adjustment				
Alogliptin	6.5–25 mg/day	No dose adjustment		12.5 mg/day	6.5 mg/day	
**GLP-1 receptor agonists (subcutaneously)**						
Exetanide	10μg/12 hours	No dose adjustment			Avoid	
Exetanide LP	2 mg/week	No dose adjustment			Avoid	
Lixisetanide	20μg/day	No dose adjustment			Avoid	
Albiglutide	30–50 mg/week	No dose adjustment			Avoid	
Dulaglutide	0.75–1.5 mg/week	No dose adjustment			Avoid	
Liraglutide	1.2–1.8 mg/day	No dose adjustment			Avoid	
Semaglutide	0.25–1 mg/week	No dose adjustment			Avoid	
**SGLT2 inhibitors (oral)**						
Empagliflozin	10–25 mg/day	No dose adjustment			Avoid	
Canagliflozin	100–300 mg/day	No dose adjustment			Avoid	
Dapagliflozin	5–10 mg/day	No dose adjustment			Avoid	
